# Comparison of the Type and Severity of Nasal Septal Deviation between Chronic Rhinosinusitis Patients Undergoing Functional Endoscopic Sinus Surgery and Controls

**DOI:** 10.1155/2022/2925279

**Published:** 2022-04-25

**Authors:** Nafiseh Nikkerdar, Atena Karimi, Fatemeh Bazmayoon, Amin Golshah

**Affiliations:** ^1^Department of Oral and Maxillofacial Radiology, School of Dentistry, Kermanshah University of Medical Sciences, Kermanshah, P.O. Code: 6715847141, Iran; ^2^Students Research Committee, School of Dentistry, Kermanshah University of Medical Sciences, Kermanshah, P.O. Code: 6715847141, Iran; ^3^Department of Orthodontics, School of Dentistry, Kermanshah University of Medical Sciences, Kermanshah, P.O. Code: 6715847141, Iran

## Abstract

**Objectives:**

Some correlations have been proposed between chronic rhinosinusitis (CRS) and type and severity of nasal septal deviation. This study sought to compare the type and severity of nasal septal deviation between CRS patients undergoing functional endoscopic sinus surgery (FESS) and asymptomatic controls using cone-beam computed tomography (CBCT).

**Materials and Methods:**

This prospective case-control study evaluated 49 CRS patients who did not respond to pharmaceutical therapy and were candidates for FESS and 49 asymptomatic controls. All participants underwent CBCT and were inspected for septal deviation type and severity. Data were analyzed by the independent *t*-test and chi-square test.

**Results:**

The study population comprised of 58.25% males and 41.8% females, with a mean age of 33.74 ± 11.78 years. Significant correlations were noted between the presence of CRS and severity of septal deviation (*P*=0.007). Type of septal deviation had no significant correlation with the presence of CRS (*P*=0.443).

**Conclusion:**

Patients with CRS have significantly more severe nasal septal deviation. However, type of septal deviation is not correlated with CRS.

## 1. Introduction

Rhinosinusitis is a common condition worldwide, imposing a significant burden on the healthcare systems. It is among the most common reasons for antibiotic prescription, and its proper management is highly important considering the emerging resistance to antibiotics [1]. Chronic rhinosinusitis (CRS) refers to complex inflammation of the nose and paranasal sinuses. Despite medical care, its signs and symptoms often last for about 12 weeks or longer [2]. CRS (with or without nasal polyps) in adults is defined as the presence of two or more symptoms, one of which should be either nasal blockage/obstruction/congestion or nasal discharge (anterior/posterior nasal drip) ± facial pain/pressure ± reduction or loss of smell for ≥12 weeks [1]. CRS is a significant health problem that affects 5–12% of the general population [1]. It also affects as high as 10.9% of the population of Europe [3]. The prevalence of sinusitis is 53% in Iran [4]. Patients with CRS often have a much lower health-related quality of life compared with healthy controls [5], and their health status has been claimed to be comparable to that of cancer, arthritis, and asthmatic patients [6].

Treatment of CRS is costly for both patients and the healthcare system. In the United States, the total cost of healthcare related to CRS was estimated to be 6.9–9.9 billion dollars/year in 2014 [7]. In case of failure of pharmaceutical therapy, functional endoscopic sinus surgery (FESS) is often performed for treatment of CRS [8]. An endoscope is used in FESS instead of open surgery. This procedure aims to clean the paranasal sinuses under direct vision while preserving the physiological health of the sinuses and the nasal cavity [9]. Nasal septal deviation can impair the normal nasal physiology. Also, it can narrow the middle meatus by moving the nasal concha laterally [10]. A recent study showed septal deviation resulting in a concavity on the other side of the septum and narrowing of the contralateral side [11]. In addition to nasal obstruction, septal deviation can obstruct the path of nasal drainage and impair the mucociliary clearance, leading to nasal congestion and secondary infection of the sinuses [10]. Nasal septal deviation has been implicated in CRS by mechanical outflow obstruction, impairment of the mucociliary function, decreasing access for surgical management, and complicating postoperative care [12–14]. Some studies have suggested that CRS is correlated with nasal septal deviation [15, 16], but some others claim that there is no correlation [17, 18]. This controversy is in need of further investigation.

Currently, medical computed tomography (CT) is the gold standard for diagnosis of many problems of the ear, nose, and throat areas [19]. Cone-beam computed tomography (CBCT) is the most important radiographic modality for three-dimensional imaging of the hard tissues. Fast technological advancements with regard to improved image quality, reproducibility, and decreased patient radiation dose have contributed to the popularity of CBCT as an alternative to medical CT and conventional radiography [20, 21]. To the best of the authors' knowledge, studies on the correlation of the severity of nasal septal deviation and its types with CRS are limited [15]. Thus, this study sought to compare the severity of nasal septal deviation and its types in CRS patients undergoing FESS versus asymptomatic controls using CBCT.

## 2. Materials and Methods

### 2.1. Study Design and Ethical Approval

The method of this study was approved by the Ethics Committee of Kermanshah University of Medical Sciences (IR.KUMS.REC.1398.199), and all procedures were performed in accordance with the ethical standards of the 1964 Declaration of Helsinki and its later versions. Written informed consent was obtained from all participants prior to their enrollment.

### 2.2. Study Population and Eligibility Criteria

This prospective case-control study evaluated 49 CRS patients undergoing FESS and 49 controls between 18 and 50 years. The CRS patients were selected among those presenting to a private ear, nose, and throat clinic to undergo FESS. The patients underwent CBCT prior to their surgical procedure. Control individuals were selected among those presenting to a private oral and maxillofacial radiology clinic to obtain CBCT scans for purposes other than the ear, nose, and throat problems.

The inclusion criteria were as follows: confirmed diagnosis of CRS according to the European Position Paper on Rhinosinusitis and Nasal Polyps (EPOS), 2020, characterized by two or more symptoms, one of which should be either nasal blockage/obstruction/congestion or nasal discharge (anterior/posterior nasal drip): ± facial pain/pressure ± reduction or loss of smell, the patients had to have this condition for more than 12 weeks with no response to medical and pharmaceutical therapy [22], and patients who had indication for medical imaging (CT/CBCT) and FESS. Patients also had mucosal changes within the ostiomeatal complex and/or sinuses on their CT scans. The exclusion criteria for CRS patients were sinus malignancies, pregnancy, immunodeficiency, cystic fibrosis, age <17 years, history of FESS, cardiac disease, rhinoplasty, and history of trauma to the face or sinuses [23].

The inclusion criterion for the controls was requiring a CBCT of the nasal and paranasal areas for purposes other than the ear, nose, and throat problems. The exclusion criteria for the controls were the same as those for the CRS patients plus any signs or symptoms in the nose, ear, and sinus areas and any history of chronic diseases in these areas. The eligible individuals were selected by convenience sampling.

### 2.3. Study Description

CBCT scans (coronal, axial, and sagittal planes) of the paranasal sinuses were obtained by the NewTom VGI CBCT scanner (QR srl, Verona, Italy) with a flat panel detector, 360-degree rotation, 14-bit signal grey scale, 3.6–5.4-second emission scan duration, a spatial resolution of 300 *μ*m (0.3 mm voxel size), a 15 × 15 cm field of view, and 110 kVp voltage.

A checklist was used to record the type and severity of septal deviation in each participant based on the CBCT scans. Two experienced oral and maxillofacial radiologists who were blinded to the group allocation of participants filled out the checklists. The CBCT scans were displayed on an LCD monitor (ASUS VS197DE, ASUS, ASUSTeK Computer Inc., Taiwan) with 1366 × 768 resolution using the NNT Viewer software version 6.1 (QR srl, Verona, Italy). To determine the severity of nasal septal deviation, a cross-sectional image on which the septum had maximum deviation was saved in Digimizer version 5.3.5 (MedCalc Software Ltd., Ostend, Belgium).

The severity of nasal septal deviation was determined by measuring the degree of septal deviation (the angle between the crista galli and the most prominent point of deviation in the coronal plane). Accordingly, the severity of septal deviation was categorized as follows [24]:

  Mild: 0°–9°
  Moderate: 10°–15°
  Severe: >15°

**Figure 1:**
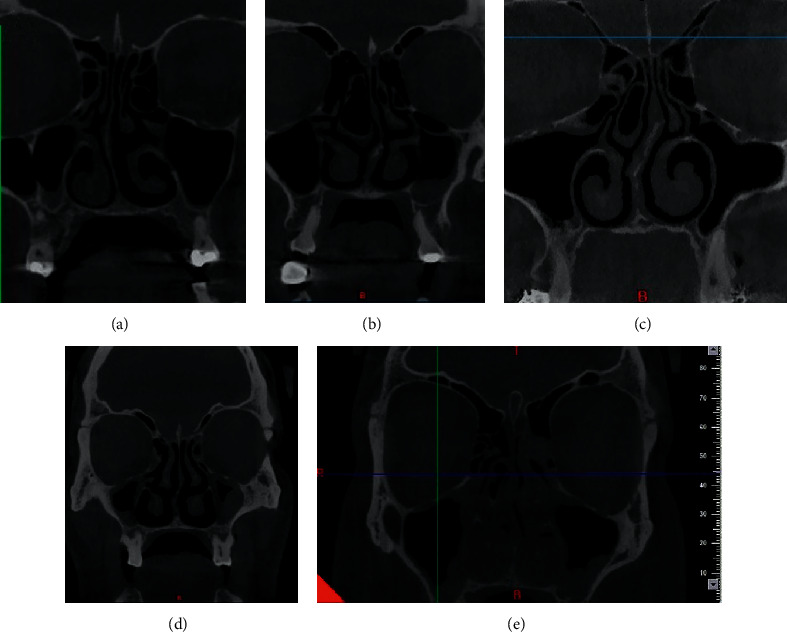
Types of septal deviation: (a) C-shaped in the coronal plane, (b) reverse C-shaped in the coronal plane, (c) reverse S-shaped in the coronal plane, (d) reverse S-shaped in the coronal plane, and (e) S-shaped in the coronal plane.

Nasal septal deviation was also divided into three main types as shown in Figure 1 (in the coronal and axial planes) [25].

### 2.4. Sample Size Estimation

The minimum sample size was calculated to be 21 in each group according to a previous study [26], assuming the effect size of 0.713, alpha = 0.05, a level of significance of 0.05, and study power of 90% using PASS (version 11; NCSS, Kaysville, UT, USA). To increase the reliability of the results, 49 patients were enrolled in each group.

### 2.5. Interexaminer Reliability

To assess the intraexaminer reliability, the CBCT scans were reevaluated again after 2 weeks, and to evaluate the interexaminer reliability between the radiologists, the intraclass correlation coefficient (ICC) was calculated for the severity of nasal septal deviation, and the kappa score was computed for the type of nasal septal deviation.

### 2.6. Statistical Analysis

The data were entered into SPSS for Windows version 20 (SPSS Inc., IL, USA) by a third person who was blinded to the group allocation of patients. Normal distribution of data was evaluated by the Kolmogorov–Smirnov test. Since the data were normally distributed, the severity of nasal septal deviation was compared between the two groups using the independent sample *t*-test. The chi-square test was applied to analyze the correlation between the qualitative variables. Level of significance was set at 0.05.

## 3. Results

The minimum sample size was calculated to be 21 in each group, but 49 patients were enrolled in each group to ensure the reliability of the results.

Of all participants, 41 (41.8%) were females and 57 (58.2%) were males. The mean age of the participants was 33.74 ± 11.78 years (range 22–46 years).

The intraexaminer (ICC = 0.963, kappa score = 0.86) and interexaminer reliability coefficients were found to be excellent (ICC = 0.952, kappa score = 0.89). The total duration of the study was approximately 12 months.

Table 1 provides the severity of septal deviation in the two groups. A significant correlation existed between the severity of nasal septal deviation and presence of CRS, such that septal deviation was significantly more severe in the CRS group compared with the control group (chi-square test, *P*=0.007).

The mean degree of nasal septal deviation was 13.97 ± 4.92 degrees in the CRS group and 10.77 ± 4.12 degrees in the control group. The CRS and control groups had a significant difference in the mean degree of nasal septal deviation, such that the mean degree of nasal septal deviation was significantly greater in the CRS group (independent samples *t*-test, *P*=0.001).

Table 2 provides the frequency of different types of nasal septal deviation in the CRS and control groups. Type of septal deviation in the horizontal plane had no significant correlation with the presence of CRS (chi-square test, *P*=0.443). The frequency of S-shaped deviation in the control group was higher than that in the CRS group, while the frequency of C-shaped deviation was higher in the CRS group, but not significantly (*P* > 0.05).

## 4. Discussion

The nasal airway is influenced by the degree, location, and type of nasal septal deviation [27]. This study sought to compare the type and severity of nasal septal deviation between CRS patients undergoing FESS and controls using CBCT. The results showed that the severity of nasal septal deviation was significantly greater in CRS patients who were candidates for FESS compared with controls. However, no other significant correlations were noted between CRS and type of nasal septal deviation.

Taghiloo and Halimi [28] reported a significant correlation between the severity of nasal septal deviation and increased mucosal thickness of the maxillary sinuses. Kumar et al. [27] reported that nasal septal deviation would make the nasal mucosa susceptible to chronic inflammation and squamous metaplasia. These changes can make the patients susceptible to CRS [27]. Gregurić et al. [17] stated that only severe nasal septal deviation had a significant correlation with the severity of sinusitis. Poorey and Gupta [29] demonstrated that increased angle of septal deviation further increased the changes in the maxillary sinus mucosa. Also, they showed that increased angle of septal deviation increased the prevalence and severity of CRS. Thus, it may be stated that more severe septal deviations can increase the risk of CRS.

In contrast to the abovementioned findings, Verhoeven and Schmelzer [30] discussed that the severity of nasal septal deviation cannot predict the severity of nasal congestion, which is a symptom of CRS. This controversy can be due to the differences in the methods of assessment and quantification of the severity of septal deviation since Verhoeven and Schmelzer [30] measured this parameter clinically while application of CT would be more appropriate for this purpose [21]. In this study, CBCT was used due to its advantages over CT.

C-shaped and reverse C-shaped deviations were the most common types of septal deviations in this study and previous investigations [31, 32]. The nasal airway and probably the discharge of mucosa into the nasal cavity are influenced by the type of nasal septal deviation [27, 33]. In a study by Alharethy et al. [33], most patients with normal discharge had S-shaped or reverse S-shaped deviations, while the most common types of septal deviation in patients who were candidates for surgery were C-shaped and reverse C-shaped deviations. Prasad et al. [34] observed that patients with C-shaped and reverse C-shaped deviations were more susceptible to sinusitis because a C-shaped septum can cause involvement of the space below the lateral superior nasal cartilage and upper surface of the inferior concha. The current results indicated the higher frequency of C-shaped and reverse C-shaped deviations in the CRS group and higher frequency of S-shaped and reverse S-shaped deviations in the control group; however, these differences were not statistically significant. This finding may be due to our small sample size, which was a limitation of this study.

Considering the current findings and those of Janovic et al. [35] and Verhoeven and Schmelzer [30], no significant correlation exists between the type of septal deviation and nasal congestion. Thus, it appears that classification (type) of septal deviation is not useful for prediction of the symptoms of CRS. Further studies are recommended on a larger sample size from different racial and ethnic groups to acquire a better perspective of the issue.

One limitation of this study was that only CRS patients who were candidates for surgery were enrolled. Future studies are required to compare the type and severity of nasal septal deviations between CRS patients not eligible for surgery and asymptomatic controls.

## 5. Conclusion

Patients with CRS have significantly more severe nasal septal deviation. However, the type of septal deviation is not correlated with CRS.

## Figures and Tables

**Table 1:** Severity of septal deviation in the two groups.

		Group				*P* value^†^
		CRS		Control		
		Count	Column *N* (%)	Count	Column *N* (%)	
Severity of nasal septal deviation	Mild	8	16.3	22	44.9	0.007
	Moderate	21	42.9	16	32.7	
	Severe	20	40.8	11	22.4	

^†^Chi-square.

**Table 2:** Frequency of different types of nasal septal deviation in the coronal and axial planes in the CRS and control groups.

		Group				*P* value^†^
		CRS		Control		
		Count	Column *N* (%)	Count	Column *N* (%)	
Types of nasal septal deviation	S-shaped	8	16.3	10	20.4	0.443
	Reverse S-shaped	5	10.2	10	20.4	
	C-shaped	22	44.9	17	34.7	
	Reverse C-shaped	14	28.6	12	24.5	

^†^Chi-square.

## Data Availability

The data used to support the findings of this study are available from the corresponding author upon request.
